# Translation and validation of the Short HIV Stigma scale in Brazilian Portuguese

**DOI:** 10.1186/s12955-020-01571-1

**Published:** 2020-10-02

**Authors:** Paula M. Luz, Thiago S. Torres, Celline C. Almeida-Brasil, Luana M. S. Marins, Daniel R. B. Bezerra, Valdilea G. Veloso, Beatriz Grinsztejn, Daphna Harel, Brett D. Thombs

**Affiliations:** 1 Instituto Nacional de Infectologia Evandro Chagas, Fundação Oswaldo Cruz, Av. Brasil 4365, Manguinhos, Rio de Janeiro, 21040-900 Brazil; 2 Research Institute of the McGill University Health Centre, Montreal, Canada; 3 Department of Applied Statistics, Social Science, and Humanities, New York University, New York, NY USA; 4 Center for Practice and Research and the Intersection of Information, Society, and Methodology, New York University, New York, NY USA; 5 Lady Davis Institute for Medical Research, Jewish General Hospital, Montréal, Québec Canada; 6 Departments of Psychiatry; Epidemiology, Biostatistics and Occupational Health; Medicine; Psychology; Educational and Counselling Psychology, and Biomedical Ethics Unit, McGill University, Montréal, Québec Canada

**Keywords:** HIV-related stigma, HIV/AIDS, Validation, Psychometric properties, Portuguese, Brazil

## Abstract

**Background:**

HIV-related stigma, or the degree to which people living with HIV endorse negative stereotypes associated with HIV, is associated with poor continuum of care outcomes. We translated the 12-item Short HIV Stigma scale and evaluated its psychometric properties in a Brazilian context with regard to construct validity and reliability.

**Methods:**

The first step included translation, back-translation, evaluation, peer review, and pre-testing of the Short HIV Sigma scale developed by Reinius et al. (Health Qual Life Outcomes 15(1):115, 2017). The second step involved piloting the scale in three convenience samples of adults recruited online through advertisements on different platforms: Grindr (October/2019) and Hornet (February–March/2020), geospatial network apps for sexual encounters for gay, bisexuals and other men who have sex with men, and social media apps (Facebook and WhatsApp, October/2019). The psychometric evaluation included confirmatory factor analysis, differential item functioning using the Multiple-Indicator Multiple-Cause model, and correlations between subscale scores and antiretroviral treatment use and adherence. Reliability was assessed using Cronbach’s alpha, and ordinal alpha and omega from the polychoric correlation matrix.

**Results:**

In total, 114, 164, and 1824 participants completed the measure items through Grindr, social media, and Hornet, respectively. We confirmed a 4-factor structure with factors for personalized stigma (3 items), disclosure concerns (3 items), concerns with public attitudes (3 items), and negative self-image (3 items). Small differential item functioning with respect to sample was found for one item (“I feel guilty because I have HIV”), which did not substantively influence estimates of latent factor scores. Grindr and Hornet’s participants scored significantly higher than social media participants on all factors except personalized stigma. Higher subscale scores correlated with antiretroviral treatment use among participants from Hornet and with lower treatment adherence in participants from Grindr and Hornet. Reliability as measured by Cronbach’s alpha, ordinal alpha and omega were 0.83, 0.88 and 0.93 for the entire scale.

**Discussion:**

The Brazilian Portuguese version of the Short HIV Stigma scale had satisfactory psychometric properties with present results suggesting that scores from different samples may be compared without concern that measurement differences substantively influence results though further studies with greater representation of women and heterosexual men are warranted.

## Background

As the HIV epidemic continues to spread in Brazil, the cumulative number of individuals with HIV/AIDS is reaching 1 million in 2020. Brazil’s HIV epidemic has, since its onset, been concentrated in key populations such as sex workers; people who use drugs; gay, bisexual and other men who have sex with men (GBM); and transgender people [1]. Indeed, though the epidemic is classified as stable at the national level, with prevalence of 0.4% in the general population [2], HIV prevalence is significantly higher in these key populations [2]. Moreover, prevalence varies geographically with a marked spatial–temporal expansion of the HIV epidemic from the major cities of the Southeast to the other regions [3].

Over the past three decades, Brazil’s response to the HIV epidemic has been strong, often leading the way when compared to other low- and middle-income countries. There are important inequities in the system, however. Black race, lower education, residing in a less developed region (most notably the North and Northeast), and high levels of social vulnerability are all independently associated with a higher likelihood of presenting to care with more advanced disease, not using antiretroviral therapy, and not achieving viral suppression [4]. An important driver of these findings may be the different forms of stigma and discrimination that are prevalent in the Brazilian society [5].

Among GBM, different forms of stigma, including internalized, perceived, experienced, and layered stigmas have been shown to significantly impact health outcomes related to HIV [6]. In Brazil, a study conducted in 2008–2009 in a national sample of GBM from 10 cities reported that 16% of participants experienced lifetime sexual violence and that the strongest predictor of sexual violence was homophobic discrimination [7]. In another analysis, concerns about confidentiality and fear of stigma and discrimination impacted HIV testing frequency [8]. A study conducted in Bahia in 2010–2011 further suggests how stigma of same-sex behavior might contribute to people living with HIV presenting to care with advanced disease [9]. Beyond sexual minority stigma, a study conducted in Belo Horizonte observed that black persons had more than 50% higher odds of experiencing discrimination than white persons in health care settings, even after controlling for income, education, social status, and health problems [10]. A call has been made for the study of the convergence of multiple stigmatized identities [11] which, for people living with HIV, will need to include the measuring and impact of HIV-related stigma.

HIV-related stigma may be broadly defined as the degree to which people living with HIV endorse negative stereotypes associated with HIV [12]. Over the past two decades, studies have shown how HIV-related stigma may affect health outcomes [13, 14]. A 2016 meta-analysis of 64 studies conducted mostly in developed countries (none in Brazil) showed that HIV-related stigma was associated with higher levels of depression and lower levels of social support, antiretroviral adherence, and access to and usage of health and social services [13]. A 2017 meta-analysis of studies from low- and middle-income countries (only one study from Brazil which evaluated HIV-stigma using a single question), suggested that HIV-related stigma doubled the odds of presenting to care with advanced disease [14]. In another study among older GBM, authors reported that, among multiple health outcomes explored, HIV-related stigma correlated with the greatest number of factors, including depression, loneliness, and substance use [15].

In Brazil, to date, few studies addressing HIV-related stigma and its impact on health and well-being have been published. In a qualitative content analysis, authors reported that people living with HIV abstain from treatment due to fear of being identified as HIV-infected in the health care setting and facing subsequent discrimination [16]. In a quantitative analysis of 900 individuals with HIV, the authors found a negative correlation between HIV-related stigma and reported physical health [17], while in a study conducted only among women, HIV-related stigma was associated with non-disclosure of HIV status to sexual partners [18]. Finally, a study conducted among 918 women in São Paulo found no association between women’s report of stigma in the context of intimate relationships and sexual inactivity [19]. It is important to note that all these studies used different instruments to measure HIV-related stigma and none described the translation and/or validation process of the instruments used.

In March 2019 Brazil joined the UNAIDS and the Global Network of People Living with HIV global assessment of HIV-related stigma (launched in 2008), and applied the stigma index version 2.0 (an 80-item instrument) in a snowball sample of 2000 individuals. Results showed how 82% of participants find it difficult to disclose their HIV-infection to others with 76% affirming that they deliberately hide their status [20].

To study HIV-related stigma, a valid and reliable instrument is needed to measure its multiple dimensions [21]. Multiple instruments are available for measuring HIV-related stigma, including full and shortened versions [22–27]. One such instrument is the Berger et al. 40-item HIV Stigma Scale, developed in the United States based on literature and two rounds of content review and psychometric analysis [28]. It measures three different stigma mechanisms as represented in the four dimensions: enacted stigma with the personalized stigma dimension, anticipated stigma with public attitudes and disclosure concerns dimensions, and internalized stigma with negative self-image dimension. The items are statements that a person living with HIV can agree or disagree with using a 4-point Likert-type response. Scoring is based on the summing of the scores for the items belonging to each subscale, or all 40-items for an overall stigma score. The Berger et al. HIV Stigma Scale had its psychometric properties evaluated in the Swedish context with regards to construct validity and reliability showing satisfactory results [29] (it has also been translated into other languages [29–32]). Aiming for a significantly shorter instrument, the Berger et al. instrument was later shortened to a 12-items Short HIV Stigma scale in the Swedish context [12]. The shorter instrument is similarly composed of statements with a 4-points Likert-type response and scoring is calculated by summing the 3 items belonging to each of the four subscales meaning that although much shorter in length, it still captures the multiple dimensions of stigma.

In the present study, we performed a translation of the Short HIV Stigma scale to Brazilian Portuguese. In addition, we evaluated its reliability (internal consistency) and construct validity in three convenience samples, each recruited online using different platforms. Furthermore, we tested for differential item functioning (DIF) to determine if any items of the scale had different measurement properties among the groups included in our sample. Items with DIF may be considered to be assessing the target construct plus some additional characteristics that differ among assessed groups. HIV affects distinct population groups that may experience HIV-related stigma differently. The presence of substantive DIF could threaten the validity of group comparisons.

## Methods

## Step 1: Translation

Following established guidelines [33], translation of the items of the Short HIV Stigma scale into Brazilian Portuguese was performed by three independent translators (two researchers and a linguistics professor fluent in both languages), after which a meeting was held to discuss and reach a consensus translated version of the scale. Then, three additional independent reviewers (two language teachers and one professional translator) translated the Portuguese version back to English, after which another meeting was held with the six members of the translation team and a mediator who was also a member of the research team to compare the original items with the back-translated items and identify where items or words seemed to differ. In this final meeting, the team reached an agreed-on version based on the comments, the original items, and the translated items. Next, three experts evaluated the translated items vis-à-vis the original subscales to judge if, in their opinion, they captured the concepts as defined. Finally, a qualitative pretesting of the resulting items was conducted with a small convenience sample to ensure item comprehensibility before moving into the second step of this study. For this, an electronic version of the scale was provided online to a sample of the target group population. Participants were requested to judge the clarity of each item on a scale from 0 to 10; if an item was scored as 7 or lower, an additional open text field was provided and the participant was asked to state what was unclear and to provide suggestions to improve clarity. A group meeting of the research team was held to discuss the suggestions made and items were adjusted as needed to improve understanding.

## Step 2: Psychometric evaluation

### Study design

This is a methodological study with a cross-sectional design that accessed the reliability and construct validity of the translated Short HIV Stigma scale in convenience samples recruited online in Brazil.

### Study population

Three convenience samples of adult Brazilians were recruited to complete a web-based survey through advertisements on different platforms. During the month of October 2019, the Grindr platform, a geospatial network app for sexual encounters for GBM, was used to recruit GBM whereas social media apps (Facebook and WhatsApp) were used to recruit women and men. During the months of February and March 2020, the Hornet platform, another geospatial network app for sexual encounters for GBM, was used to recruit a second sample of GBM.

For the web-based survey launched in October 2019, given its significant length, random allocation of eligible participants to different instruments was performed to decrease participant burden such that each participant only responded to one instrument. The web-based survey launched in February and March 2020 on Hornet, instead, was reduced in length and did not use random allocation, with participants responding to all survey items applicable to them.

**Fig. 1:**
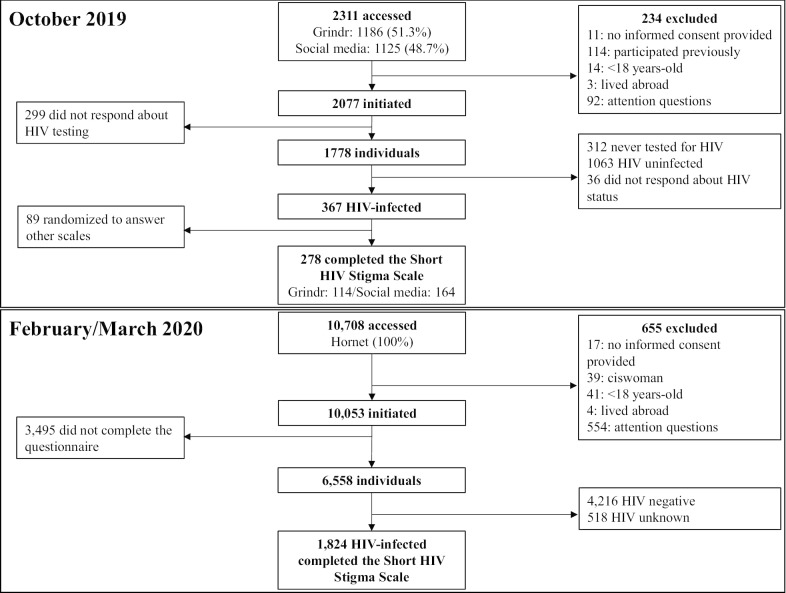
Study population flow-chart for the two web-based surveys launched in October 2019 and February/March of 2020

Participant eligibility included age ≥ 18 years, residency in Brazil, and self-report of HIV-infection. Exclusion criteria was an incorrect response to any of the attention questions which were included throughout the survey instrument at approximately every 15 items, and having responded to the survey previously (Fig. 1: Participant flow chart). This study was approved by INI Evandro Chagas-FIOCRUZ institutional review board (#CAAE 01777918.0.0000.5262) in accordance with all applicable regulations, and participants provided informed consent prior to being directed to survey items.

### Study instrument and measurements

Relevant to the present study, the survey instrument was divided into four sections as follows. Section 1 included items on socio-demographic information (age, race/ethnicity, gender, sexual orientation, education, income, state of residence), and Sect. 2 included items referring to prior HIV testing and HIV test results. If HIV-infected, participants answered Sect. 3 which included the items of the translated scale. As in the original scale [12], response options for the translated scale were on a 4-point Likert ranging from strongly disagree (*discordo totalmente*, 1) to strongly agree (*concordo totalmente*, 4), and scale scoring was calculated by summing the items of each subscale (subscale range: 3–12).

Section 4 included items on use of antiretroviral treatment (Are you currently using antiretroviral therapy? Yes/No), and if in use, treatment adherence. Treatment adherence was measured using the WebAd-Q instrument, a 3-items web-based questionnaire developed from interview and focus groups with people living with HIV in Brazil and subsequently validated in a sample of 74 individuals using antiretroviral treatment for more than 60 days [34]. The items constitute questions (i.e. In the prior 7 days, did you forget to take any of your prescribed pills?) that should be answered as Yes, No, or I don’t remember. Scoring is based on the sum of “Yes” responses with adherence defined as a “No” response to all three items. The WebAd-Q instrument was shown to correlate with other measures of adherence (pill count, electronic monitoring and self-report) and with subsequent viral load measurements [34]. Additionally, we also assessed adherence with the question “Please mark below the value that corresponds to how much of your antiretroviral medication you took in the past 30 days?” which was answered on slider using a visual analog scale and adherence was defined using the usual cut-off of 95% or more [35].

## Statistical analysis

Socio-demographic characteristics were described with absolute counts and percentages.

### Confirmatory factor analysis

We first tested the original factor structure of the Short HIV Stigma scale in the three samples separately using confirmatory factor analysis (CFA). Item responses were ordinal Likert data, so the weighted least squares estimator with a diagonal weight matrix, robust standard errors, and a mean- and variance-adjusted chi-square statistic was used with delta parameterization [36]. Modification indices were inspected to identify pairs of items within scales for which model fit would improve if error estimates were freed to covary [37]. To assess model fit, chi-square test, Tucker-Lewis Index (TLI) [38], Comparative Fit Index (CFI) [39], Root Mean Square Error of Approximation (RMSEA) [40], and Standardized Root Mean Square Residual (SRMR) [41] were used. Since chi-square test is highly sensitive to sample size, it can lead to the rejection of well-fitting models [42]. Therefore, the TLI, CFI and RMSEA fit indices were emphasized. Good fitting models were indicated by a TLI and CFI ≥ 0.95 and RMSEA ≤ 0.06 [43].

Once the factor structure was established for each sample separately, a CFA model was fit that included participants from two distinct sample populations. As previously described, GBM carry the greatest HIV burden in Brazil with a pattern of increasing incidence in the past decade. Moreover, HIV-infected GBM, as sexual minorities, suffer from other forms of stigma suggesting that they may experience HIV-related stigma differently. Thus, to reach our objective of assessing for the presence of substantive DIF which could threaten the validity of group comparisons, for the analyses that follow, both samples of GBM (Grindr and Hornet) were compared to social media participants separately.

### Differential item functioning

To determine whether Short HIV Stigma Scale items exhibited DIF for Grindr versus social media participants and for Hornet versus social media participants, we followed a pre-established protocol using a multiple indicator multiple cause (MIMIC) model [44–46]. These are models where the relationship between factors and a set of covariates are studied to understand measurement invariance and population heterogeneity. The base MIMIC model consists of the CFA factor model with an added direct effect of group on the latent factors. This serves to control for group differences on the latent factor. Then, each item is separately regressed on group to assess potential DIF, defined as a statistically significant (*p* < 0.05) link of group with the item, controlling for differences in the overall level of the latent factor. Once all items with significant DIF were identified, the magnitude of DIF items collectively was evaluated by comparing the difference on the latent factor between groups in the baseline model and after controlling for DIF.

### Reliability

We estimated each subscale’s internal consistency using Cronbach’s alpha and assumed it acceptable if > 0.7 [47]. Additionally, we also estimated two ordinal reliability coefficients, ordinal alpha and omega, from the polychoric correlation matrix [48].

### Construct validity

We used hypothesis testing (Wilcoxon Signed Rank Test for Grindr and social media samples and Student’s *t*-test for Hornet sample) to evaluate if subscale scores of the Short HIV Stigma scale differed by use of antiretroviral treatment or treatment adherence for each group separately. We hypothesized that higher scores on the Short HIV Stigma subscales would be associated with a decreased use of antiretroviral treatment and decreased adherence.

CFA and MIMIC analyses were carried out in Mplus version 8.4, all other analyses were performed in R version 4.0.2 [49].

## Results

### Step 1: Translation

The qualitative pretesting of the final translated version was done with a small convenience sample of the target group population (N = 11), most were aged 25–35 years (45%), had finished basic education (45%), and lived in the city of Rio de Janeiro (73%). On a scale of 0 to 10, all items were judged as clear by most participants with a lowest mean clarity score of 8.3 for item 3 (“Some people avoid touching me”) and a highest of 10 for item 6 (“I am very careful who I tell”). Two items, 5 and 11, were slightly modified as a function of the suggestions made by participants. The final version of the translated scale is given in Table 1.

**Table 1:** The 12 final items of the translated short HIV stigma scale

Item#	Original item	Final Brazilian Portuguese translation
	Personalized stigma	
01	People I care about stopped calling after learning I have HIV	Pessoas que eu gosto pararam de falar comigo quando souberam que eu tenho HIV
02	I have lost friends by telling them I have HIV	Já perdi amigos depois de contar que tenho HIV
03	Some people avoid touching me once they know I have HIV	Algumas pessoas evitam me tocar depois que descobrem que tenho HIV
	Disclosure concerns	
04	I work hard to keep my HIV a secret	Me esforço para manter em segredo que tenho HIV
05	Telling someone I have HIV is risky	Tenho receio de contar para alguém que tenho HIV
06	I am very careful who I tell that I have HIV	Tomo muito cuidado com quem falo que tenho HIV
	Concerns about public attitudes	
07	Most people believe a person who has HIV is dirty	A maioria das pessoas acredita que quem tem HIV é sujo
08	People with HIV are treated like outcasts	Pessoas com HIV são marginalizadas
09	Most people are uncomfortable around someone with HIV	A maioria das pessoas se sente desconfortável na presença de alguém com HIV
	Negative self-image	
10	I feel guilty because I have HIV	Me sinto culpado por ter HIV
11	People’s attitudes about HIV make me feel worse about myself	As atitudes das pessoas em relação ao HIV fazem com que me sinta mal
12	I feel I’m not as good a person as others because I have HIV	Sinto que não sou uma pessoa tão boa quanto as outras por ter HIV

### Step 2: Psychometric evaluation

#### Sample characteristics

Socio-demographic characteristics for the three study samples are displayed in Table 2.

**Table 2:** Characteristics of the three study samples

	Grindr	Social media	Hornet
Total	114	164	1824
Age			
Mean (SD)	38.6 (10.1)	43.7 (12.3)	37.8 (9.9)
Age categories			
18–24	8 (7.0)	6 (3.7)	103 (5.6)
25–29	16 (14.0)	21 (12.8)	301 (16.5)
30–39	39 (34.2)	38 (23.2)	721 (39.5)
40–49	33 (28.9)	42 (25.6)	423 (23.2)
50–59	16 (14.0)	38 (23.2)	238 (13.0)
60+	2 (1.8)	19 (11.6)	38 (2.1)
Gender			
Cisgender men	114 (100)	89 (54.3)	1794 (98.4)
Cisgender women	0 (0)	63 (38.4)	0 (0)
Transgender/non-binary	0 (0)	12 (7.3)	30 (1.6)
Sexual orientation			
Gay/homosexual	104 (91.2)	87 (54.0)	1665 (91.4)
Bisexual	10 (8.8)	6 (3.7)	146 (8.0)
Heterosexual	0 (0)	68 (42.2)	9 (0.5)
Skin color/race			
White	63 (56.8)	75 (46.3)	1121 (63.0)
Black	14 (12.6)	25 (15.4)	191 (10.7)
Pardo (Mixed)/native	34 (30.6)	62 (38.3)	467 (26.3)
Income^a^			
Low	26 (22.8)	93 (56.7)	497 (27.2)
Middle	57 (50.0)	58 (35.4)	832 (45.6)
High	31 (27.2)	13 (7.9)	495 (27.1)
Education (years)^b^			
≤ 12	30 (26.5)	100 (61.0)	565 (31.4)
> 12	83 (73.5)	64 (39.0)	1233 (68.6)
Region^c^			
Other	36 (31.6)	27 (16.5)	121 (6.6)
Southeast/South	78 (68.4)	137 (83.5)	1703 (93.4)
Partner			
No	83 (72.8)	82 (50.0)	–
Yes	31 (27.2)	82 (50.0)	–
Short HIV Stigma Scale scores			
Personalized stigma			
Mean (SD)	5.9 (2.9)	5.9 (2.8)	5.5 (2.5)
Disclosure concerns			
Mean (SD)	11.1 (1.5)	8.9 (3.2)	10.6 (2.1)
Concerns about public attitudes			
Mean (SD)	9.8 (1.9)	8.7 (2.4)	9.3 (2.2)
Negative self-image			
Mean (SD)	7.8 (2.6)	6.7 (2.5)	7.3 (2.6)

^a^We considered the number of minimum wages in the family monthly income: low ≤ 2, middle > 2–6, high > 6 (monthly minimum wage in 2019 was 998 BRL = US $248, currency from January 2020)

^b^ ≤ 12 years is equivalent to complete Secondary Education or less, > 12 is equivalent to complete College education or higher

^c^Other = North, Northeast and Central-west regions

– Data not available

*Grindr platform* In total, 114 cisgender men completed the translated version of the Short HIV Stigma scale. Median age was 38 years (interquartile range 31–46), most were white (63, 57%), 31 (27%) reported high-income, and 83 (73%) reported college education or higher (more than 12 years of education). Sexual orientation was reported as gay/homosexual by 104 (91%) and bisexual by 10 (9%). Subscale scores were 5.9 (SD 2.9) for personalized stigma, 11.1 (SD 1.5) for disclosure concerns, 9.8 (SD 1.9) for concerns about public attitudes, and 7.8 (SD 2.6) for negative self-image.

*Social media platforms* In total, 164 participants completed the translated version of the Short HIV Stigma scale. Median age was 43 years (interquartile range 33–54), 75 (46%) were white, 93 (57%) reported low income, and 64 (39%) reported college education or higher. The majority were cisgender men (87, 54%) and 12 (7%) were transgender/non-binary. Subscale scores were 5.9 (SD 2.8) for personalized stigma, 8.9 (SD 3.2) for disclosure concerns, 8.7 (SD 2.4) for concerns about public attitudes, and 6.7 (SD 2.5) for negative self-image.

*Hornet platform* In total, 1,824 participants completed the translated version of the Short HIV Stigma scale (98.4% cisgender men and 1.6% transgender/non-binary). Median age was 36 years (interquartile range 30–43), most were white (1121, 63%), 495 (27%) reported high-income, and 1233 (67%) reported college education or higher (more than 12 years of education). Sexual orientation was reported as gay/homosexual by 1665 (91%) and bisexual by 146 (8%). Subscale scores were 5.5 (SD 2.5) for personalized stigma, 10.6 (SD 2.1) for disclosure concerns, 9.3 (SD 2.2) for concerns about public attitudes, and 7.3 (SD 2.6) for negative self-image.

Comparing the samples that include exclusively GBM, Grindr and Hornet (Table 2), we note important similarities in age, gender and sexual orientation. There were small differences in income and education (greater representation of lower income and less educated in the Hornet sample), and larger differences in the spatial distribution of participants: 93% of those using Hornet are from the South/Southeast. Comparing the two samples of GBM with social media participants, we note that participants from social media were older, reported lower education and income, and were more likely to have a partner (50% social media vs 27% Grindr). Importantly, the social media sample allowed the inclusion of multiple genders: cisgender women (38%), transgender/non-binary (7%) and cisgender men (54%), and of individuals of heterosexual orientation.

### Analysis 1: Grindr and social media samples

#### Confirmatory factor analysis

A four-factor structure showed good fit in both samples [Grindr: chi-square (48) = 56.9, *p* = 0.17, CFI = 0.995, TLI = 0.993, RMSEA = 0.040, SRMR = 0.062; social media: chi-square (48) = 63.4, *p* = 0.07, CFI = 0.993, TLI = 0.990, RMSEA = 0.044, SRMR = 0.047, Additional file 1: Tables S1 and S2]. Construct validity was supported with overall high standardized loadings of items on the intended scales, except for items 10 (0.634) and 12 (0.592) in the Grindr sample and items 8 (0.555) and 10 (0.436) in the social media sample. No modifications were made based on modification indices.

#### Differential item functioning

The four-factor model was fit to the combined samples (Grindr and social media), including a direct effect of group on the latent factors. Results indicate a good fit [chi-square (56) = 72.6, *p* = 0.07, CFI = 0.995, TLI = 0.993, RMSEA = 0.033, SRMR = 0.038]. Prior to accounting for possible DIF, Grindr participants had 0.02 SD lower personalized stigma factor levels than social media participants (*p* = 0.87). For the three other factors, Grindr participants had significantly higher factor levels: 0.97 SD higher for disclosure concerns, 0.49 SD higher for concerns about public attitudes, and 0.25 SD higher in negative self-image than social media participants (*p* < 0.01 for all, Table 3). DIF analyses indicated that only item 10 (“I feel guilty that I have HIV”) was significant, being endorsed at higher levels by Grindr participants (0.30 SD higher than social media participants, *p* = 0.02).

**Table 3:** Factor loadings for the Short HIV Stigma scale in the base and DIF corrected models and influence of group on the overall estimates of latent factor scores: analysis 1 includes Grindr and social media samples and analysis 2 includes Hornet and social media samples

Short HIV stigma items	Analysis 1: Grindr and social media samples		Analysis 2: Hornet and social media samples	
	Base model	DIF corrected	Base model	DIF corrected
Personalized stigma				
People I care about stopped calling after learning I have HIV	0.896	0.896	0.854	0.854
I have lost friends by telling them I have HIV	0.883	0.883	0.901	0.901
Some people avoid touching me once they know I have HIV	0.853	0.853	0.860	0.860
Disclosure concerns				
I work hard to keep my HIV a secret	0.945	0.945	0.900	0.900
Telling someone I have HIV is risky	0.946	0.946	0.955	0.955
I am very careful who I tell that I have HIV	0.908	0.908	0.861	0.861
Concerns about public attitudes				
Most people believe a person who has HIV is dirty	0.839	0.839	0.839	0.839
People with HIV are treated like outcasts	0.614	0.614	0.732	0.732
Most people are uncomfortable around someone with HIV	0.745	0.745	0.813	0.813
Negative self-image				
I feel guilty because I have HIV	0.529	0.503	0.677	0.673
People’s attitudes about HIV make me feel worse about myself	0.891	0.895	0.872	0.872
I feel I’m not as good a person as others because I have HIV	0.662	0.660	0.670	0.670
Structural effect of group on latent factor^a^				
Personalized stigma	− 0.020	− 0.020	− *0.145*	− *0.145*
Disclosure concerns	*0.969*	*0.969*	*0.647*	*0.647*
Concerns about public attitudes	*0.487*	*0.487*	*0.243*	*0.243*
Negative self-image	*0.252*	*0.194*	*0.180*	0.120
Direct effect on item attributable to group				
I feel guilty because I have HIV (item 10)		0.298		*0.219*

^a^Social Media group is reference

Italics indicate *p* value < 0.01

As shown in Table 3, after correcting for DIF for item 10, compared with the base model, there was a decrease of 0.06 SD on the latent negative self-image factor for Grindr participants compared to social media, a negligible difference with no differences on the other factors. Thus, although there was statistically significant DIF on item 10, this did not substantively influence the overall estimates of HIV stigma latent factor scores between the two samples. After correcting for DIF, Grindr participants scored 0.97, 0.49 and 0.19 SD higher on the latent factors disclosure concerns, concerns about public attitudes, and negative self-image, respectively, than social media participants.

### Analysis 2: Hornet and social media samples

#### Confirmatory factor analysis

A four-factor structure showed good fit in the Hornet sample [chi-square (54) = 498.2, *p* < 0.01, CFI = 0.980, TLI = 0.973, RMSEA = 0.071, SRMR = 0.039, Additional file 1: Table S3]. Construct validity was supported with overall high standardized loadings of all items (ranging from 0.671 for item 12 to 0.958 for item 5).

#### Differential item functioning

The four-factor model was fit to the combined samples (Hornet and social media), including a direct effect of group on the latent factors. Results indicate a good fit [chi-square (56) = 483.6, *p* < 0.01, CFI = 0.983, TLI = 0.976, RMSEA = 0.06, SRMR = 0.034]. Prior to accounting for possible DIF, Hornet participants had 0.14 SD lower personalized stigma factor levels than social media participants (*p* = 0.05). For the three other factors, Grindr participants had significantly higher factor levels: 0.65 SD higher for disclosure concerns, 0.24 SD higher for concerns about public attitudes, and 0.18 SD higher in negative self-image than social media participants (*p* < 0.01 for all, Table 3). DIF analyses indicated that only item 10 (“I feel guilty that I have HIV”) was significant, being endorsed at higher levels by Hornet participants (0.22 SD higher than social media participants, *p* < 0.01).

After correcting for DIF for item 10, compared with the base model, there was a decrease of 0.06 SD on the latent negative self-image factor for Hornet participants compared to social media, a negligible difference with no differences on the other factors. Thus, although there was statistically significant DIF on item 10, this did not substantively influence the overall estimates of HIV stigma latent factor scores between the two samples. After correcting for DIF, Hornet participants scored 0.65, and 0.24 SD higher on the latent factors disclosure concerns and concerns about public attitudes, respectively, than social media participants.

### Reliability

Considering all samples combined, reliability as measured by Cronbach’s alpha, ordinal alpha and omega were, respectively, 0.85, 0.91 and 0.91 for personalized stigma; 0.87, 0.93, and 0.94 for disclosure concerns; 0.76, 0.84, and 0.84 for concerns about public attitudes; 0.69, 0.78, 0.78 for negative self-image; and 0.83, 0.88 and 0.93 for the entire scale.

### Construct validity

Results showed that participants reached by the Grindr platform who were not on antiretroviral treatment scored higher than those on treatment, no differences were observed for the social media sample and, for Hornet participants, those on antiretroviral treatment scored higher than those not on treatment (Table 4). A more consistent correlation was found between antiretroviral treatment adherence and subscale scores. Among the three samples and the two adherence measures, participants who self-reported non-adherent on the Web-Ad-Q and the visual analog scale scored higher than those who were adherent on personalized stigma. Additionally, among participants from Hornet, those who were non-adherent scored higher with respect to concerns about public attitudes and negative self-image.

**Table 4:** Mean (standard deviation) of Short HIV Stigma sub-scale scores as a function of antiretroviral therapy initiation and treatment adherence in Grindr, Social Media, and Hornet samples

	Antiretroviral treatment use^a^				Adherence^b^				Adherence^c^			
	No	Yes	Test statistic^d^	p^d^	No	Yes	Test statistic^d^	p^d^	No	Yes	Test statistic^d^	p^d^
Grindr, N (%)	4 (3.5)	110 (96.5)			39 (35.5)	71 (64.5)			14 (12.7)	96 (87.3)		
Personalized stigma	9.5 (3.0)	5.7 (2.8)	365	0.02	6.7 (3.0)	5.2 (2.6)	998	0.01	5.0 (2.3)	5.9 (2.9)	768	0.38
Disclosure concerns	12.0 (0.0)	11.1 (1.5)	296	0.16	11.1 (1.5)	11.1 (1.6)	1402	0.90	11.1 (1.5)	11.1 (1.6)	687	0.87
Concerns about public attitudes	11.0 (1.4)	9.8 (1.9)	304	0.19	10.1 (1.9)	9.6 (2.0)	1176	0.19	9.4 (1.6)	9.8 (2.0)	802	0.24
Negative self-image	8.0 (3.7)	7.8 (2.6)	231	0.87	8.0 (2.4)	7.6 (2.6)	1286	0.54	7.3 (2.2)	7.8 (2.6)	757	0.45
Social media, N (%)	6 (3.7)	158 (96.3)			70 (45.2)	85 (54.8)			20 (12.9)	135 (87.1)		
Personalized stigma	4.5 (2.0)	6.0 (2.8)	326	0.19	6.6 (3.0)	5.6 (2.5)	2392	0.03	8.8 (2.3)	5.6 (2.6)	507	< 0.01
Disclosure concerns	8.8 (3.2)	8.9 (3.2)	433	0.72	8.8 (3.2)	9.1 (3.1)	3125	0.58	9.8 (2.2)	8.9 (3.3)	1248	0.58
Concerns about public attitudes	8.5 (3.4)	8.7 (2.4)	487	0.85	8.8 (2.5)	8.6 (2.3)	2705	0.32	8.9 (2.2)	8.6 (2.4)	1270	0.69
Negative self-image	7.5 (3.3)	6.6 (2.5)	551	0.50	6.8 (2.7)	6.6 (2.3)	2899	0.78	7.2 (2.9)	6.6 (2.4)	1202	0.31
Hornet, N (%)	78 (4.3)	1746 (95.7)			741 (42.4)	1005 (57.6)			247 (14.1)	1499 (85.9)		
Personalized stigma	5.4 (2.7)	5.5 (2.5)	0.27	0.79	5.7 (2.6)	5.4 (2.5)	2.36	0.02	5.9 (2.7)	5.5 (2.5)	2.45	0.01
Disclosure concerns	8.4 (3.6)	10.7 (1.9)	9.68	< 0.01	10.7 (1.9)	10.6 (1.9)	0.90	0.37	10.7 (1.8)	10.7 (1.9)	0.36	0.72
Concerns about public attitudes	8.5 (2.6)	9.3 (2.2)	3.1	< 0.01	9.5 (2.2)	9.3 (2.2)	1.93	0.05	9.7 (2.1)	9.3 (2.2)	2.49	0.01
Negative self-image	6.8 (2.8)	7.3 (2.6)	1.49	0.14	7.6 (2.6)	7.1 (2.5)	4.17	< 0.01	7.8 (2.7)	7.2 (2.5)	3.53	< 0.01

^a^ “Have you started antiretroviral treatment for your HIV-infection?” Possible options Yes/No

^b^Validated instrument (WebAd-Q, Vale et al. 2017) composed of three questions: In the prior 7 days, (1) did you take any of your pills at a different time than the prescribed scheduled time, (2) did you forget to take any of your prescribed pills, and (3) did you take more or less of your prescribed pills. Response options include Yes/No/I don’t remember. Adherence was defined as a “No” to all three questions

^c^A visual analog scale was provided along with a question “Please mark below the value that corresponds to how much of your antiretroviral medication you took in the past 30 days.” Responses were coded as adherence if participants selected ≥ 95%

^d^Test statistic and corresponding p-values for Wilcoxon Signed Rank Test for Grindr and social media samples and Student’s t-test for Hornet sample

^*^Adherence items are applicable to those on treatment and for whom data was not missing (see Fig. 1)

## Discussion

In this study, we employed multiple steps and techniques to translate and validate the Short HIV Stigma scale in Brazilian Portuguese. Our results represent a first assessment of the Short HIV Stigma scale in this population and future studies are needed to expand our results. Nonetheless, we found that the Brazilian Portuguese version of the Short HIV Stigma scale had satisfactory psychometric properties and construct validity was supported by the correlations between subscale scores and the use of and adherence to antiretroviral treatment. Of particular importance is the possible occurrence of differential item functioning that “can be conceptualized as a form of measurement bias, where individuals respond to items on a scale as a function of some attribute other than what the scale is designed to measure” [50]. We found significant differential item functioning of negligible magnitude (SD = 0.06) for one item addressing feelings of guilt (“I feel guilty because I have HIV”). As such, our findings suggest that the Short HIV Stigma scale may be used in different populations in Brazil and that score comparisons can be made without concern that measurement differences may substantively influence results.

Although our results did not evidence DIF of relevant magnitude between groups, we did observe large differences in the levels of the construct. Grindr and Hornet participants scored significantly higher on the latent factors disclosure concerns and concerns about public attitudes, and ~ 0.20 SD higher on the latent factor negative self-image, than social media participants. In the absence of DIF, these would appear to be actual differences in the three of the four dimensions of the construct being measured (HIV-related stigma). HIV-infected GBM are prone to other forms of stigma including homonegativity, or the negative attitudes a person has towards his own homosexuality [51]. In a study of older (aged 50–65 years) HIV-infected GBM, authors reported how HIV stigma coupled with two other forms of stigma (homonegativity and ageism) explained 39% of the variance associated with self-reported quality of life [51]. The stereotype of HIV as a disease of gay men coupled with Brazil’s heterosexist and anti-gay society could lead to a higher HIV-related stigma among GBM. Moreover, as suggested [52], these societal-level conditions and cultural-norms may act synergistically and more severely affect the physical and mental health of GBM.

There are several study limitations to highlight. Our three samples were recruited from different web-based platforms, and there was a difference in sample size between the samples. As such, the core model from analysis 1 used to assess DIF relied more on data from social media participants than those from Grindr, while for analysis 2, it relied more on data from Hornet participants than those from social media. Since the initial confirmatory factor analysis yielded similar model structures for all samples, it does not seem likely that the different sample sizes influenced results substantially. Due to the cross-sectional design of the study, test–retest reliability and sensitivity to change of the translated Short HIV Stigma scale could not be assessed. Also, participants were recruited from convenience samples and not through a random sampling of people living with HIV. It is possible that our participants vary systematically from the entire population of people living with HIV, and this may have impacted our findings and decreased the generalizability of our results. Compared to people living with HIV in Brazil [1], our sample was older, had less women, and was mostly from the Southeast/South.

## Conclusions

Studies are needed for a more accurate and realistic understanding of how HIV-related stigma hinders Brazil’s response to the HIV epidemic. A validated instrument to measure HIV-related stigma should empower studies to evaluate its impact on the well-being of populations. We hope that the availability of a validated Short HIV Stigma scale will foster studies in Brazil using a standardized instrument, thus allowing for better comparison across studies. Indeed, we believe that this short instrument will be particularly useful in a scenario where multiple additional constructs could be measured and their interplay explored.

## Supplementary information

**Additional file 1.** Supporting information for manuscript by Luz et al. Translation and validation of the Short HIV Stigma Scale in Brazilian Portuguese.

## Availability of supporting data

The datasets used and/or analyzed during the current study are available from the corresponding author on reasonable request.
