# Supplementary material for: Translation and validation of the Short HIV Stigma scale in Brazilian Portuguese
Source: Health Qual Life Outcomes. 2020 Oct 2;18:322. doi: 10.1186/s12955-020-01571-1 (PMC7530962; doi:10.1186/s12955-020-01571-1)
Supplement: Supplementary file 1 — Additional file 1. Supporting information for manuscript by Luz et al. Translation and validation of the Short HIV Stigma Scale in Brazilian Portuguese. [file 12955_2020_1571_MOESM1_ESM.docx]

**Additional file 1**

**Table S1. Standardized factor loadings, model fit indices and internal consistency reliability coefficients of the Short HIV Stigma Scale for the Grindr sample.**

| **Grindr (N = 114)** | **Standardized loadings** | **Cronbach alpha** | **Ordinal alpha** | **Omega** |
| --- | --- | --- | --- | --- |
| **Personalized stigma** |  | 0.90 | 0.94 | 0.94 |
| People I care about stopped calling after learning I have HIV | 0.936 |  |  |  |
| I have lost friends by telling them I have HIV | 0.922 |  |  |  |
| Some people avoid touching me once they know I have HIV | 0.891 |  |  |  |
| **Disclosure concerns** |  | 0.81 | 0.93 | 0.94 |
| I work hard to keep my HIV a secret | 0.943 |  |  |  |
| Telling someone I have HIV is risky | 0.958 |  |  |  |
| I am very careful who I tell that I have HIV | 0.824 |  |  |  |
| **Concerns about public attitudes** |  | 0.65 | 0.76 | 0.76 |
| Most people believe a person who has HIV is dirty | 0.810 |  |  |  |
| People with HIV are treated like outcasts | 0.693 |  |  |  |
| Most people are uncomfortable around someone with HIV | 0.667 |  |  |  |
| **Negative self-image** |  | 0.63 | 0.72 | 0.73 |
| I feel guilty because I have HIV | 0.634 |  |  |  |
| People’s attitudes about HIV make me feel worse about myself | 0.813 |  |  |  |
| I feel I’m not as good a person as others because I have HIV | 0.592 |  |  |  |
| **12-items** |  | 0.79 | 0.87 | 0.93 |
| **Model fit indices** |  |  |  |  |
| Chi-square (48) = 56.9, p = 0.17 |  |  |  |  |
| CFI = 0.995 |  |  |  |  |
| TLI = 0.993 |  |  |  |  |
| RMSEA = 0.040 (90%CI 0.000-0.076) |  |  |  |  |
| SRMR = 0.062 |  |  |  |  |

**Table S2. Standardized factor loadings, model fit indices and internal consistency reliability coefficients of the Short HIV Stigma Scale for the social media sample.**

| **Social media (N = 164)** | **Standardized loadings** | **Cronbach alpha** | **Ordinal alpha** | **Omega** |
| --- | --- | --- | --- | --- |
| **Personalized stigma** |  | 0.83 | 0.88 | 0.89 |
| People I care about stopped calling after learning I have HIV | 0.868 |  |  |  |
| I have lost friends by telling them I have HIV | 0.850 |  |  |  |
| Some people avoid touching me once they know I have HIV | 0.831 |  |  |  |
| **Disclosure concerns** |  | 0.90 | 0.95 | 0.95 |
| I work hard to keep my HIV a secret | 0.922 |  |  |  |
| Telling someone I have HIV is risky | 0.938 |  |  |  |
| I am very careful who I tell that I have HIV | 0.817 |  |  |  |
| **Concerns about public attitudes** |  | 0.69 | 0.76 | 0.78 |
| Most people believe a person who has HIV is dirty | 0.846 |  |  |  |
| People with HIV are treated like outcasts | 0.555 |  |  |  |
| Most people are uncomfortable around someone with HIV | 0.782 |  |  |  |
| **Negative self-image** |  | 0.62 | 0.73 | 0.75 |
| I feel guilty because I have HIV | 0.436 |  |  |  |
| People’s attitudes about HIV make me feel worse about myself | 0.910 |  |  |  |
| I feel I’m not as good a person as others because I have HIV | 0.710 |  |  |  |
| **12-items** |  | 0.83 | 0.87 | 0.92 |
| **Model fit indices** |  |  |  |  |
| Chi-square (48) = 63.4, p = 0.07 |  |  |  |  |
| CFI = 0.993 |  |  |  |  |
| TLI = 0.990 |  |  |  |  |
| RMSEA = 0.044 (90%CI 0.000-0.071) |  |  |  |  |
| SRMR = 0.047 |  |  |  |  |

**Table S3. Standardized factor loadings, model fit indices and internal consistency reliability coefficients of the Short HIV Stigma Scale for the Hornet sample.**

| **Hornet (N = 1824)** | **Standardized loadings** | **Cronbach alpha** | **Ordinal alpha** | **Omega** |
| --- | --- | --- | --- | --- |
| **Personalized stigma** |  | 0.85 | 0.90 | 0.91 |
| People I care about stopped calling after learning I have HIV | 0.853 |  |  |  |
| I have lost friends by telling them I have HIV | 0.906 |  |  |  |
| Some people avoid touching me once they know I have HIV | 0.863 |  |  |  |
| **Disclosure concerns** |  | 0.86 | 0.93 | 0.93 |
| I work hard to keep my HIV a secret | 0.891 |  |  |  |
| Telling someone I have HIV is risky | 0.958 |  |  |  |
| I am very careful who I tell that I have HIV | 0.854 |  |  |  |
| **Concerns about public attitudes** |  | 0.77 | 0.84 | 0.85 |
| Most people believe a person who has HIV is dirty | 0.838 |  |  |  |
| People with HIV are treated like outcasts | 0.750 |  |  |  |
| Most people are uncomfortable around someone with HIV | 0.819 |  |  |  |
| **Negative self-image** |  | 0.69 | 0.79 | 0.79 |
| I feel guilty because I have HIV | 0.698 |  |  |  |
| People’s attitudes about HIV make me feel worse about myself | 0.865 |  |  |  |
| I feel I’m not as good a person as others because I have HIV | 0.671 |  |  |  |
| **12-items** |  | 0.83 | 0.88 | 0.93 |
| **Model fit indices** |  |  |  |  |
| Chi-square (48) = 489.2, p < 0.01 |  |  |  |  |
| CFI = 0.980 |  |  |  |  |
| TLI = 0.973 |  |  |  |  |
| RMSEA = 0.071 (90%CI 0.065-0.077) |  |  |  |  |
| SRMR = 0.039 |  |  |  |  |
